# Editorial: Neuro-Education and Neuro-Rehabilitation

**DOI:** 10.3389/fpsyg.2016.01427

**Published:** 2016-09-22

**Authors:** Eduardo Martínez-Montes, Julie Chobert, Mireille Besson

**Affiliations:** ^1^Neuroinformatics Department, Cuban Neuroscience CenterLa Habana, Cuba; ^2^Laboratoire de Neurosciences Cognitives, UMR 7291, Centre National de la Recherche Scientifique, Aix-Marseille UniversitéMarseille, France

**Keywords:** learning disorders, language, musical training, sensorimotor training, neurofeedback

The latest advances in cognitive psychology, neuropsychology, and cognitive neuroscience has brought from science fiction to reality the possibility of influencing our brain activity (Owen et al., 2010; Glannon, 2014; Gruzelier, 2014a). Better understanding of brain functioning and brain plasticity has allowed neuroscientists to transfer findings from fundamental research to education and to the rehabilitation of learning disabilities (Besson et al., 2011; Goswami, 2016). The emerging fields of neuro-education and neuro-rehabilitation aim at creating effective and safe programs to improve brain functioning related to specific perceptive, cognitive, emotional, and motor abilities. Some attempts to achieve these goals take advantage of the use of natural mechanisms, such as those mediating the interactions between brain and arts (Särkämö et al., 2008; Bringas et al., 2015). Others use experimental designs to make the brain aware of its own activity, creating the so-called neurofeedback loop (Gruzelier, 2014b). Succeeding in these goals would constitute an achievement of high societal impact (Davidson and McEwen, 2012; Vuilleumier et al., 2014).

This *Frontiers Research Topic* brings together 16 articles that cover a broad scope of topics in the relatively young but very dynamic fields of neuro-education and neuro-rehabilitation. Contributed by world-renowned scientists in cognitive psychology, neuropsychology, and cognitive neuroscience, often experts in different types of learning disorders, this E-book is organized around three main themes: neuro-education, neuro-rehabilitation and basic research with relevance to both fields. Each theme includes Review articles covering the state-of-the-art of knowledge in a specific sub-domain, Original Research articles reporting new discoveries and Opinion and Hypothesis and Theory articles adding exciting new ideas and approaches for neuro-educational and neuro-rehabilitation methods.

In the first part, dedicated to neuro-education, Ylinen and Kujala review the impact of auditory or phonological training on the level of performance in various tasks and on the neural basis of behavior in children with dyslexia, children with specific language impairment and children with language-learning impairment. François et al. review the efficacy of musical training for language learning. They highlight several studies showing that learning to play a musical instrument can induce substantial neuro-plastic changes in cortical and subcortical regions of motor, auditory and speech processing networks. They show evidence that musical training can be an alternative, low-cost and effective method for the treatment of language-learning impaired populations, as well as for patients with stroke or Parkinson Disease. Direct support for the use of musical training for the rehabilitation of children with dyslexia is reported by Habib et al. who tested the efficacy of a specially-designed Cognitivo-Musical training (CMT) method. Intensive short-term CMT with dyslexic children yielded significant improvements in categorical and auditory perception of temporal components of speech, while long-term CMT provided additional improvements in auditory attention, phonological awareness, reading abilities and repetition of pseudo words. Along the same lines, Fonseca-Mora et al. also tested the efficacy of a new phonological training program (with and without music), for teaching to read in a foreign language, demonstrating its beneficial effects on early reading skills but without additional improvements linked to music training. Final but not least, Kraus et al. present the results of a longitudinal study examining the impact of a community music program on language development in children from low socio-economic backgrounds. Children more engaged in the music program developed stronger brain encoding of speech and improved reading scores, thereby suggesting that this kind of program provides children with auditory enrichments that may counteract some of the biological consequences of growing up in poverty. To conclude this section, the reader will find a novel approach to neuro-education, as proposed by Gerdes et al. They view learners in terms of their neurodevelopmental trajectories and propose a groundwork for allostatic neuro-education (GANE). Illustrative case studies of the use of GANE in children with Asperger's syndrome, attention-deficit hyperactivity disorder and reading difficulties are presented.

The second part of this E-book is dedicated to neuro-rehabilitation. Serniclaes et al. review the efficacy of remediation methods that tap into core deficits in dyslexia (phonemic, grapho-phonemic, and graphemic) and examine how some of these methods may contribute to the remediation of allophonic perception. Guilbert et al. describe different procedures for sensory training in unilateral spatial neglect (USN) and present recent scientific evidence that makes music a good candidate for USN patients' rehabilitation. Dhami et al. consider the use of dancing as an intervention tool and as a potential parallel to physical and music therapies, since dancing also engages various perceptive, cognitive, emotional and motor functions. The opinion paper from Stahl and Kotz addresses three relevant issues in current research on singing and aphasia: articulatory tempo, clinical research designs and formulaic language resources. The authors discuss how these issues may reconcile seemingly contradictory findings in the literature and provide guidelines for future research based on holistic and analytic approaches that may help improving the efficacy of music-based aphasia therapy. Finally, in a Hypothesis and Theory article, Elmer and Jäncke consider the use of a neurofeedback approach for auditory rehabilitation. They first stress the advantages of using intracerebral functional connectivity (IFC) instead of quantitative EEG for interventional applications and then propose concrete interventional IFC applications that may improve auditory-related dysfunctions such as developmental dyslexia.

In the third part, we compiled some interesting studies which contribute both to a better comprehension of basic psychophysiological mechanisms and to the development of potential applications for neuro-education and neuro-rehabilitation. Vidal et al. review the role of sensorimotor information for motor learning. They discuss the effects of several factors known to influence information processing in sensorimotor activities based on the distinction between extrinsic (e.g., quantity and quality of information, level of instruction and motor program learning) and intrinsic factors (e.g., prior information, individual strategies and capabilities for fast error detection). In a more specific context, Berteletti and Booth investigated the extent to which somatosensory information from the fingers contributes to numerical sense in children. Their work provided first neurological evidence for a functional role of the somatosensory finger area in proficient arithmetic problem solving, thereby encouraging educational practices to integrate finger-based strategies as a tool for instilling stronger numerical sense. Still in another context, writing, Danna and Velay review studies that use natural sensory and supplementary feedback to help the writer learn how to write and to control writing. They discuss the role of each sensory modality, how information is used in handwriting control and how this control changes with practice and learning. Turning from writing to reading, Boudelaa reports, in an original research article, that the processing time course in auditory modality is different for consonants and vowels in Arabic. The implications of this work for neuro-education and neuro-rehabilitation in Arabic are discussed. Finally, Tandonnet et al. illustrate how basic approaches in cognitive science may benefit human factors engineering and potentially improve man-machine interfaces.

We hope this compilation of articles describing the latest research in the field of neuro-education and neuro-rehabilitation will be of interest to the readers and will impulse even more research in these fascinating new fields with strong societal impact.

## Author contributions

All authors listed, have made substantial, direct and intellectual contribution to the work, and approved it for publication.

### Conflict of interest statement

The authors declare that the research was conducted in the absence of any commercial or financial relationships that could be construed as a potential conflict of interest.
